# Hyperbaric Oxygen Therapy and A-PRF Pre-Treated Implants in Severe Periodontitis: A Case Report

**DOI:** 10.3390/ijerph18020413

**Published:** 2021-01-07

**Authors:** Tommaso Antonio Giacon, Franco Giancola, Matteo Paganini, Cesare Tiengo, Enrico M. Camporesi, Gerardo Bosco

**Affiliations:** 1 Department of Biomedical Sciences, Environmental and Respiratory Physiology, University of Padova, Via Marzolo 3, 35131 Padova, Italy; gerardo.bosco@unipd.it; 2 Clinica Europea Implantologia Ceramica, Domus Medica, 47890 Città di San Marino, San Marino; info@francogiancola.com; 3 Clinic of Plastic Reconstructive and Aesthetic Surgery, Padova University Hospital, 35128 Padova, Italy; cesare.tiengo@unipd.it; 4 TeamHealth Anesthesia, 1 Tampa General Circle, Tampa, FL 33606, USA; enrico_camporesi@teamhealth.com

**Keywords:** periodontitis, hyperbaric oxygen therapy, A-PRF, implantology, dental implants, hyperbaric, dentistry, bone regeneration

## Abstract

Implantation is currently the best option for tooth replacement in periodontitis. Some major contraindications for the immediate implant are acute periodontitis and active infection. We present the case of a 51-year-old female patient with the highest grade and stage periodontitis treated with advanced platelet-rich fibrin-enriched zirconia implants and with hyperbaric oxygen therapy (HBOT). In particular, HBOT before and after implantation promoted bone regeneration and implant integration, also providing an antiseptic effect. After six months, the implants were well established and fully healed from periodontal disease within 14 months. Further research could confirm a new indication for HBOT in treating periodontitis and dental implantation.

## 1. Introduction

Hyperbaric oxygen therapy (HBOT) consists of breathing high oxygen concentrations at pressures that exceed 1 atm abs (101.325 kPa). Its efficacy is obtained by enhancing reactive oxygen species (ROS) and reactive nitrogen species (RNS) production, promoting cell growth, and modulating inflammatory response. As a result, vascularization and post-ischemic tissue survival is significantly improved [1,2]. Its effects on chronic wounds, namely the prevention and contrast of infections caused by anaerobic pathogens on soft tissues, are well known [3,4,5,6]. HBOT also promotes bone regeneration by improving osteosynthesis, neoangiogenesis, and vasculogenesis. It has been successfully used in many pathologies characterized by osteonecrosis, such as avascular necrosis of the femoral head (AVNFH) and osteonecrosis of the knee [7,8,9]. In particular, referring to the oral and maxillofacial districts, HBOT increased the bone regeneration rate in osteoradionecrosis, mandibular osteomyelitis, and dental implants [10,11,12,13,14,15,16,17].

Periodontitis is a chronic inflammatory disease affecting soft tissues that support and surround the teeth and bone [18]. Caused mainly by bacterial plaque and local immune response, periodontitis can also lead to loss of tooth attachment. During the disease’s progression, a change in the oral microbiota is recorded, showing a prevalence of anaerobic bacteria such as *P. gingivalis* [13]. HBOT in periodontitis was shown to improve clinical parameters such as probe depth (PD), clinical attachment level (CAL), and bleeding on probing (BOP) compared with standard antibiotic and dental medicine treatments. In particular, positive effects were registered after eight treatment sessions at >1.4 atmospheres absolute (ATA) [10,19], but specific indications on HBOT use in periodontitis are lacking.

In this case report, we describe the use of HBOT and advanced platelet-rich fibrin (A-PRF) pre-treated implants in an immediate dental implant due to severe periodontitis and loss of tooth attachment, despite strong clinical contraindications. The treatment and the report of the case were authorized by the pertaining ethical committee (B200-2020-088) and, after careful information, the patient gave written consent for both.

## 2. Case Report

**Figure 1:**
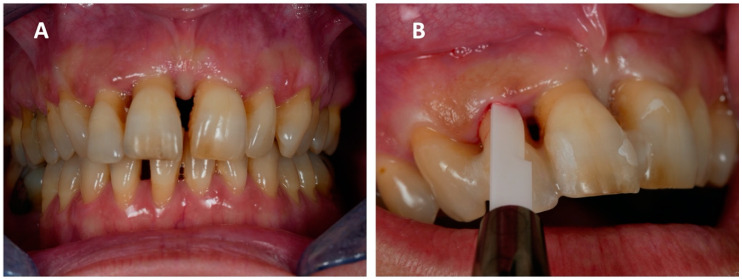
The disease’s initial status at the first visit: (**A**) Diffused and significant periodontitis can be seen, mainly affecting the superior incisors, and (**B**) deep periodontal pockets with active inflammation and bleeding.

A 51-year-old woman came to our attention due to her superior incisor pain and mobility (Universal Numbering System No. 7, 8, 9, 10). Her past medical history was unremarkable, except for being an active smoker. The dental examination found diffused mobility, deep periodontal pockets, bleeding, and suppurative discharge when probing (Figure 1A,B), posing the diagnosis of severe periodontitis. High-grade gingival recession was also reported, along with dentine hypersensitivity. Percussion to superior incisors exacerbated pain and their vestibularization caused chewing problems. Superior incisors were classified with grade 3 mobility, a PD between 5 and 8 mm, and diffused BOP (Supplementary Material Figure S1).

**Figure 2:**
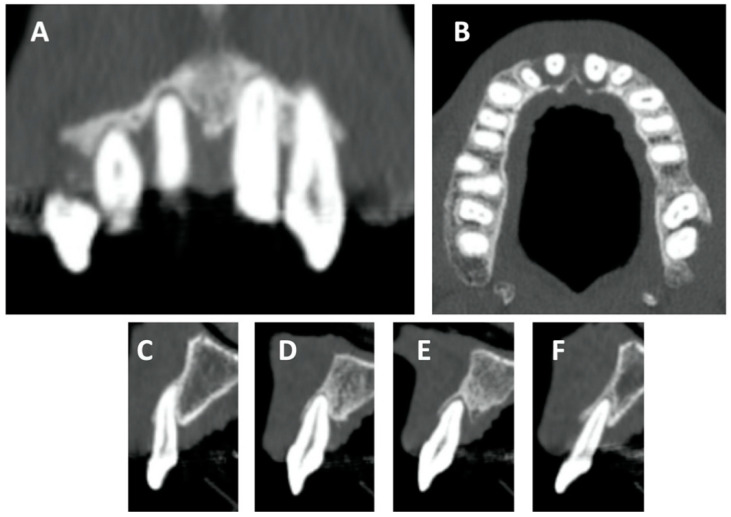
Details obtained from CBCT: (**A**) superior incisor coronal view, (**B**) superior incisor axial view, and (**C**–**F**) superior incisor sagittal view. Diffused radiolucent areas suggest a low grade of bone attachment, particularly in the area of the superior incisors.

Loss of bone attachment was radiologically documented through panoramic dental x-ray and cone beam computed tomography (CBCT), confirming the disease’s clinical features. Coronal and sagittal views of the superior incisors derived from CBCT showed a severe loss of attachment that correlated with the extreme mobility of the superior incisors (Figure 2A–F).

**Figure 3:**
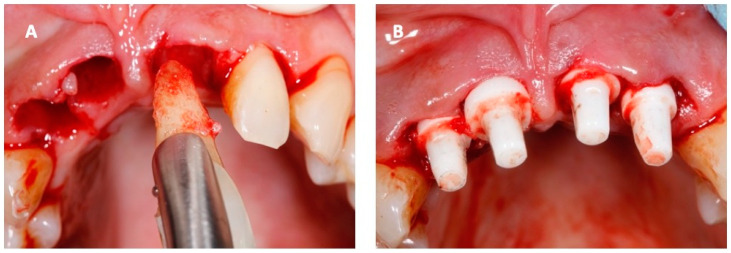
Pictures obtained the day of the operation: (**A**) atraumatic extraction of the superior incisors and (**B**) insertion of the zirconia implants in the bonified alveoli.

Periodontitis was classified as stage IV, grade C, according to the new classification of periodontal disease [20]. Acute periodontitis and suppuration are absolute contraindications to the immediate positioning of post-extraction implants. However, the patient firmly requested an immediate implant to relieve her physical and psychological discomfort. The intervention was performed in a single session in August 2019. The four superior incisors were removed with an atraumatic technique, and the implant site was prepared using piezoelectrical inserts (Piezosurgery^®^, Mectron s.p.a., Carasco, Italy). The implants were composed of zirconia (Zirkolith^®^ Z5m, Z-systems AG, Oensingen, Switzerland) previously soaked for 10 min in A-PRF obtained from autologous blood tissue of the patient by centrifugation at 1200 rpm for 10 min (Universal Centrifuge, LWScientific, USA). A-PRF was absorbed in the porous structure of the implants and provided an external coating. Then the implants were inserted with a dedicated surgical kit (Figure 3) respecting an inter-implant distance of more than 1.5 mm. At the end of the operation, some prints were obtained to create temporary resin dental crowns for functional and aesthetic purposes, then anchored to the adjacent teeth to avoid any movement and promote normal osteointegration of the implants. No immediate complications were recorded.

According to the will of the patient, no antibiotic therapy was administered.

HBOT was used to help implant integration and to prevent infectious complications. A total of 10 sessions of HBOT, 60 min daily at 2.2 ATA, were administered: three preconditioning sessions immediately before the extraction—to contrast active infection and enhance tissue vascularization—and seven after the implant. The sessions were administrated using the institution’s eight-person hyperbaric chamber (Sistemi Iperbarici s.r.l., Pomezia, Italy).

**Figure 4:**
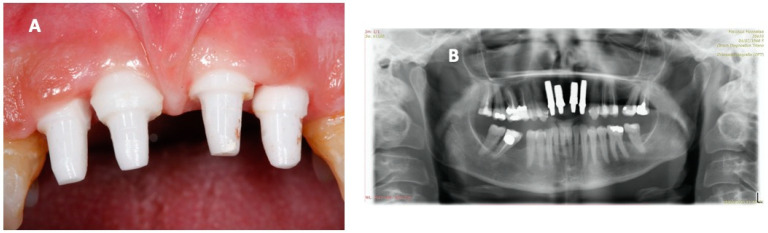
Pictures obtained six months after the operation, showing (**A**) a good state of osteointegration, no bleeding, and no active infection, with healthy periodontal tissue, and (**B**) dental radiography showing a good grade of osteointegration and no significant radiolucent areas.

The patient, aware of the disease’s severity, regularly attended the HBOT sessions, which helped soft tissue healing and osteointegration. As shown by panoramic x-ray, the clinical picture was optimal at the six-month follow-up (Figure 4). Magnetic resonance imaging (MRI) was performed at the follow-up instead of a computed tomography (CT), in order to avoid scattering artifacts and unnecessary radiation exposure. The images showed optimal osteointegration and no bone density reduction (Supplementary Material Video S2).

**Figure 5:**
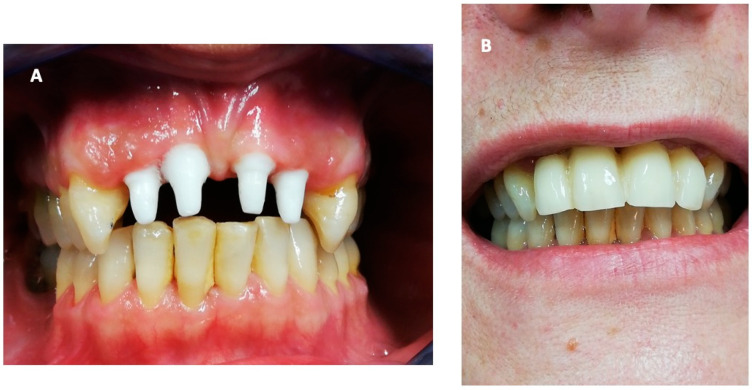
Pictures obtained 14 months after the operation: (**A**) healthy periodontal tissues and integrated implants and (**B**) the final aesthetic effect with zirconia dental crowns.

Fourteen months after the intervention, the periodontal soft tissues appeared healthy and with an optimal periodontal seal. The other teeth also showed less severe periodontitis, with improvements in CAL and BOP. The patient felt relieved from a physical and psychological perspective and reported no significant complications during the whole period. The prosthesis was then finalized with esthetic zirconia ceramic crowns (Figure 5).

## 3. Discussion

Periodontitis is a common disease characterized by extreme clinical variability causing significant difficulties in the diagnosis. Many classifications have been proposed to define the cause and to provide a severity index [21]. According to the new classification of periodontal disease, the stage (I to IV) refers to the severity, complexity, and extent of the disease (loss of attachment, bone loss, and tooth loss), whereas the grade (A to C) indicates the biological features and progression rate of the disease [20]. Smoking habits and diabetes mellitus impact the grade and induce a faster and more severe disease progression. In this severe periodontitis case, the patient had the highest grade and stadium periodontitis and was an active smoker, but quit smoking by the end of the follow-up after educational interventions helping the achievement of the results.

Implantology is currently the best treatment option for replacing missing teeth, but the presence of a healthy alveolus is crucial for its success. Acute periodontitis and infections are major contraindications due to the high risk of failure and complications such as peri-implantitis and other deep tissue or bony infections [22]. A thorough exam before suggesting an immediate implant is mandatory, and practitioners should always perform an accurate surgical debridement of the alveolus before the implant, trying to remove any infected or necrotic tissue. The patient underwent the intervention despite contraindications after sharing the plan and carefully balancing the harms and benefits, especially considering the psychological impairment due to the advanced disease.

At the moment, HBOT is not officially recommended as a treatment for periodontitis. However, HBOT is effective and indicated by the Tenth European Consensus Conference on Hyperbaric Medicine in treating osteoradionecrosis, femoral head necrosis, and complicated anaerobic or mixed bacterial infections [23], pathologies that share with periodontitis a similar background of bone loss and inflammation due to chronic infection. In the following sections, a possible rationale for using HBOT as a treatment and preconditioning in periodontitis is discussed.

In acute bone injuries, the inflammatory cascade has a key role in reparation and osteogenesis. On the other hand, chronic inflammation is known to generate high levels of pro-inflammatory markers and results in bone resorption, a situation encountered in rheumatic diseases [24]. HBOT modulates pro-inflammatory markers by paradoxically generating ROS and enhancing oxidative damage protective mechanisms. ROS play a key role in many pathways leading to neoangiogenesis and vasculogenesis mediated by hypoxia-inducible factors (HIFs) and involving vascular endothelial growth factor (VEGF) and basic fibroblast growth factor (bFGF) [1,2]. In an in vitro-reproduced chronic inflammatory model, HBOT improved the expression of osteogenic markers in mesenchymal stem cells (MSCs) and enhanced mineral deposition [7]. Treating osteoblasts with HBOT also improved their proliferation rate compared to hypoxic or normoxic conditions and increased alkaline phosphatase activity (ALP) [25]. Hyperbaric oxygen relieved radiation-induced osteonecrosis in rats by contrasting the inflamed, hypocellular, hypovascular, and hypoxic environment in the damaged bone and promoting vascularization [13,14]. In patients with AVNFH, hyperbaric oxygen was demonstrated to reduce inflammatory markers such as tumor necrosis factor-α (TNF-α) and interleukin (IL)-6, creating a favorable osteogenic environment [8]. Periodontitis provides an excellent example of bone loss due to chronic inflammation and immune response. In patients with periodontitis, inflammatory markers (IL-6, IL-10, TNF-α, C-reactive protein, and ALP) in the gingival crevicular fluid are increased and directly correlated with the severity of the disease [26]. In treating periodontitis, HBOT seemed to improve clinical parameters and reduce subgingival anaerobe growth, making it a promising adjuvant combined with standard procedures such as scaling and root planing [27,28,29]. After the implant, the patient underwent seven HBOT sessions, a number considered sufficient in current literature to achieve optimal results [19]. However, further clinical studies are needed to clarify its role in this subset of patients.

HBOT is known to inhibit bacterial growth (both aerobic and anaerobic) and improve the effects of antibiotics [30]. The combined use of antibiotics and hyperbaric oxygen showed promising results in treating complicated dog bite wounds and sepsis caused by anaerobic bacteria (*C. canimorsus*) [31] by improving the postantibiotic effect (PAE) and inhibited bacterial growth in Pseudomonas infections [31,32]. HBOT also enhances immune response efficacy by preserving lymphocyte functions and especially stimulating neutrophil defenses [33]. After hyperbaric oxygen exposure, ROS production from neutrophils’ mitochondria and myeloperoxidase transcription are significantly incremented, along with higher RNS levels due to higher expression of inducible nitric oxide synthase (i-NOS) [2]. Lack of oxygen is fundamental to favoring anaerobic infections and is crucial in biofilms. In the inner layers, the oxygen concentration is critically low, and the antibiotic effect is compromised due to the reduced formation of ROS and metabolites or drug uptake. On the contrary, hyperbaric oxygen can penetrate the deepest layers and increase antibiotic susceptibility, as previously demonstrated with *P.Aeruginosa* and *S.Aureus* biofilms [34,35,36]. Since a higher availability of ROS can facilitate neutrophils killing activity, HBOT has promising future clinical applications, especially with the constant increase of drug-resistant pathogens [37]. Biofilms characterizing many pathologies, such as dental plaque in periodontitis, are very difficult to eradicate through antibiotic therapy due to a complex organization and heterogeneity of bacteria, a different gradient of growth rate, antibiotic resistance, and metabolic activity. Hyperbaric oxygen alone has mild biofilm-killing properties but showed improved clinical results combined with antibiotics [38]. In this case, the patient refused antibiotic therapy, but hyperbaric oxygen contributed to the excellent outcome. The best option would be to use both appropriate antibiotics and HBOT to create an appropriate synergy to improve the effectiveness of the treatment and extend its range of action.

Pretreatment with HBOT has been successfully used to prevent osteoradionecrosis caused by radiotherapy for head and neck malignancies following the Marx protocol [11,39,40,41]. Hyperbaric oxygen preconditioning also prevented ischemia-reperfusion injury, particularly in myocardium and brain tissue, and other inflammatory situations [42]. In fact, HBOT pretreatment stimulates the transcription of oxidative stress protection proteins and prepares tissues for interventions entailing transient ischemia. In this severe case, a preconditioning with three sessions of HBOT improved the outcome probably through successful sterilization of the surgical site and enhancement of antioxidant defenses, but future studies should specifically address this topic.

Zirconia is a perfect material for implants since it has superior mechanical and chemical properties and biocompatibility with less inflammatory response than other metals such as titanium. Single-piece implants were applied due to fewer biological and technical complications than two-piece implants, as reported in the literature [43,44]. However, zirconia’s main drawback is the bonding difficulty that requires many treatments to increase its wetting capacity [45]. Different implant coating techniques improve osteointegration, particularly bioactive materials such as platelet-rich plasma (PRP) and platelet rich fibrin (PRF). A wound with active bleeding that produces a good fibrine clot promotes an efficient neoangiogenesis [46], and PRP and PRF amplify this natural phenomenon [47,48]. Platelet-derived growth factors (PDGF) in contact with the implant surface help osteointegration even in immediate implantation when sites are not fully healed [49,50]. Many studies support PDGF use for periodontal and soft tissue repair, but it seems to produce good results in bone regeneration and improve the success rate in immediate implantation [51]. A-PRF seems to induce a faster proliferation and healing rate than leukocyte and platelet-rich fibrin (L-PRF), fibroblast grow factor (FGF), or a negative control group [52]. The implants used in this case were soaked in A-PRF, a platelet concentrate obtained after the patient’s blood was centrifuged, which worked as a coating to help clotting, neovascularization, and osteointegration [53].

## 4. Conclusions

Despite no official indications for HBOT in treating periodontitis, its use in necrotizing soft tissue infections, wound healing, skeletal muscle-compartment syndromes, and osteoradionecrosis is well established. In this case of severe periodontitis, even without antibiotic therapy and an unfavorable prognosis due to contraindications, hyperbaric oxygen proved useful as preconditioning and adjuvant therapy to prevent infections and promote neovascularization. Moreover, HBOT facilitated implantation, promoting osteointegration and tissue regeneration. In addition, zirconia implants treated with A-PRF proved reliable and capable of excellent results in immediate implants. No side effects have been recorded in this patient despite the intensive HBOT protocol. In the future, these results should be tested through randomized trials on a larger sample, also introducing antibiotic therapy along with HBOT.

## Data Availability

All data are provided in the manuscript.

## Supplementary Materials

The following are available online at https://www.mdpi.com/1660-4601/18/2/413/s1, Figure S1: Full periodontal chart, Video S2: Maxillofacial MRI.
