# Supplementary figures and images for: Hyperbaric Oxygen Therapy and A-PRF Pre-Treated Implants in Severe Periodontitis: A Case Report
Source: Int J Environ Res Public Health. 2021 Jan 7;18(2):413. doi: 10.3390/ijerph18020413 (PMC7825644; doi:10.3390/ijerph18020413)

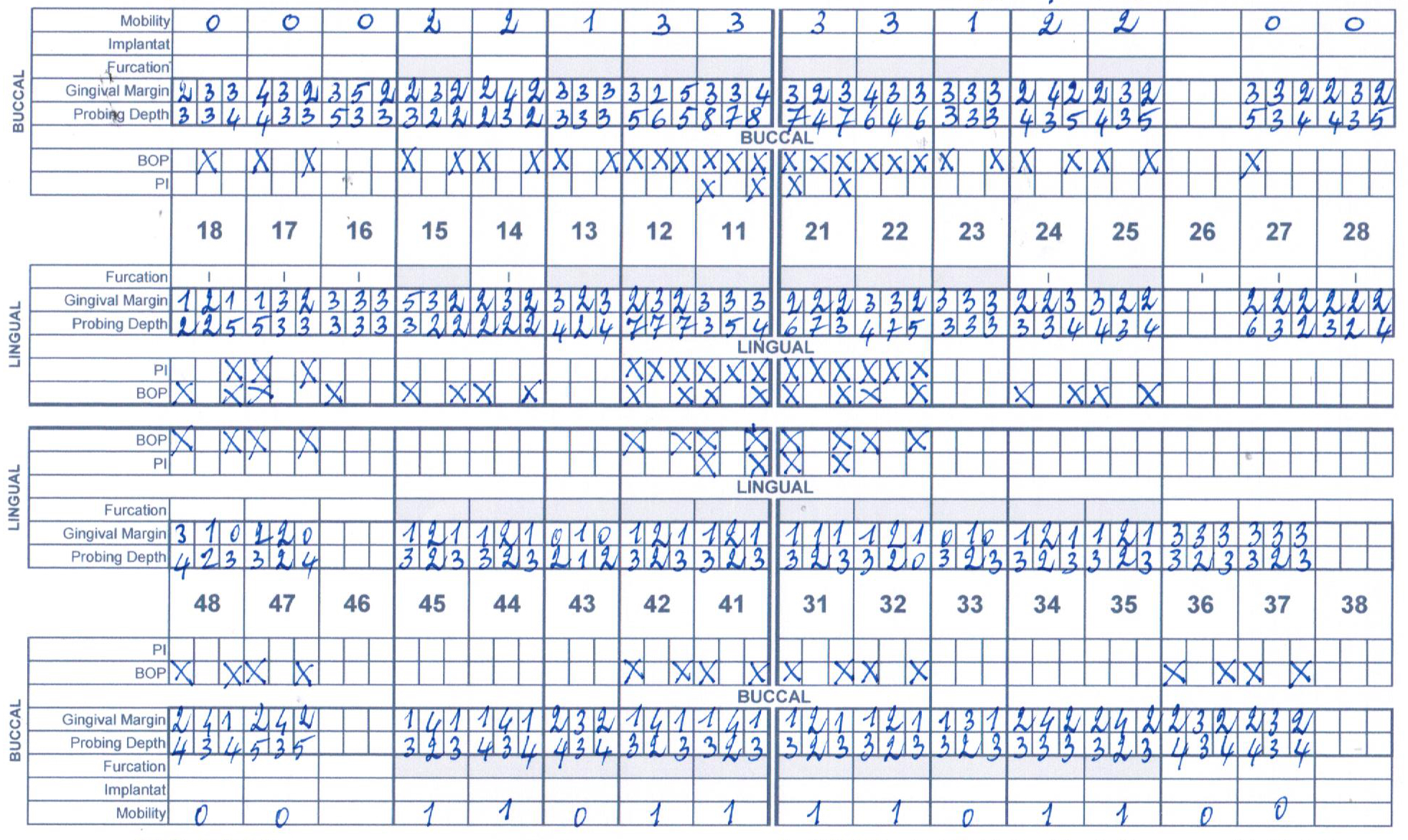

Supplement: Supplementary file 1 [file ijerph-18-00413-s001.zip › Supplementary 1, periodontal chart.jpg]
